# Untargeted Metabolite Profiling of Cerebrospinal Fluid Uncovers Biomarkers for Severity of Late Infantile Neuronal Ceroid Lipofuscinosis (*CLN2*, Batten Disease)

**DOI:** 10.1038/s41598-018-33449-0

**Published:** 2018-10-15

**Authors:** Miriam Sindelar, Jonathan P. Dyke, Ruba S. Deeb, Dolan Sondhi, Stephen M. Kaminsky, Barry E. Kosofsky, Douglas J. Ballon, Ronald G. Crystal, Steven S. Gross

**Affiliations:** 1 Department of Pharmacology, Weill Cornell Medicine, New York, New York USA; 2 Department of Radiology, Weill Cornell Medicine, New York, New York USA; 3 Department of Genetic Medicine, Weill Cornell Medicine, New York, New York USA; 4 Department of Pediatrics, Weill Cornell Medicine, New York, New York USA

## Abstract

Late infantile neuronal ceroid lipofuscinosis (*CLN2* disease) is a rare lysosomal storage disorder caused by a monogenetic deficiency of tripeptidyl peptidase-1 (TPP1). Despite knowledge that lipofuscin is the hallmark disease product, the relevant TPP1 substrate and its role in neuronal physiology/pathology is unknown. We hypothesized that untargeted metabolite profiling of cerebrospinal fluid (CSF) could be used as an effective tool to identify disease-associated metabolic disruptions in *CLN2* disease, offering the potential to identify biomarkers that inform on disease severity and progression. Accordingly, a mass spectrometry-based untargeted metabolite profiling approach was employed to differentiate CSF from normal vs. *CLN2* deficient individuals. Of 1,433 metabolite features surveyed, 29 linearly correlated with currently employed disease severity scores. With tandem mass spectrometry 8 distinct metabolite identities were structurally confirmed based on retention time and fragmentation pattern matches, vs. standards. These putative *CLN2* biomarkers include 7 acetylated species – all attenuated in *CLN2* compared to controls. Because acetate is the major bioenergetic fuel for support of mitochondrial respiration, deficient acetylated species in CSF suggests a brain energy defect that may drive neurodegeneration. Targeted analysis of these metabolites in CSF of *CLN2* patients offers a powerful new approach for monitoring *CLN2* disease progression and response to therapy.

## Introduction

*CLN2* disease, a form of Batten disease, is a rare autosomal recessive pediatric neurodegenerative disorder that arises from a lysosomal storage defect. Characteristic of *CLN2* disease is the accumulation of auto-fluorescent ceroid lipopigments in neural and peripheral tissues^1^. *CLN2* disease is characterized by a monogenetic deficiency of tripeptidyl peptidase-1 (TPP1)^2^, a lysosomal serine protease that cleaves diverse tripeptides sequentially from unprotected polypeptide N-termini^3,4^. Additionally, TPP1 functions as an endopeptidase by cleaving between hydrophobic residues^5^, which recently has been ascribed to its ability to proteolyze β-amyloid, the hallmark accumulation product in Alzheimer’s disease^6^. In the setting of *CLN2* disease, accumulated ceroid-lipofuscin predominantly includes a product of the mitochondrial ATP synthase subunit C^7^, and multiple other unprocessed neuropeptides. Despite knowing the identity of the lipopigment in *CLN2* disease, the pathological mechanism and metabolic disruptions that trigger neuronal death are unknown. Currently, the potential diagnosis of *CLN2* disease is initially based on clinical findings (i.e., retinopathy, motor abnormalities, dementia and/or epilepsy), followed by *CLN2* gene sequencing and assessment of TPP1 enzymatic activity to confirm the diagnosis^8^.

First symptoms of *CLN2* disease typically appear at 2–4 years of age (i.e., cognitive impairment, seizures, progressive vision loss and deteriorating motor skills), eventually culminating in blindness and dementia^9^. Most often, the disease is fatal by 10 to 12 years of age^10^. To monitor *CLN2* disease progression, a clinical severity score was developed at Weill Cornell Medical College (Weill Cornell *LINCL* Scale, WCLS)^11,12^. This scale is based on four parameters, each ranging in value from 0–3, and contributing equally to an additive overall score. These four parameters are feeding, motor abilities, gait, and language development, with a maximum score of 12 in the case of undetectable impairments^11^. Alternatively, the Hamburg scale, a different 12-point scale can be applied, based on motor abilities, language development, vision and seizures^12^. More recently, a whole brain magnetic resonance imaging disease severity score (MRIDSS) was developed based on the N-acetylaspartate/creatine ratio (NAA/Cre), %volume of CSF (%CSF) and the apparent diffusion coefficient (ADC). This score linearly correlates with the WCLS^13^. In the past, the only available therapies were those that ameliorate *CLN2* symptoms (e.g. epilepsy). However, in 2017 an enzyme replacement therapy using recombinant TPP1 was Food and Drug Administration (FDA)-approved^14,15^. A potential long-term gene therapy cure is currently in development, using a brain-delivered adeno-associated virus gene vector that expresses human *CLN2* cDNA (AAVrh.10hCLN2)^16^ in attempt to restore central nervous system (CNS) TPP1 activity.

Lysosomal storage diseases are known to broadly disrupt cell metabolism^17^. Accordingly, we hypothesized that *CLN2* disease results in distinct discoverable metabolic perturbations that may provide mechanistic insights in the disease pathology, and additionally provide novel biomarkers to monitor disease severity and response to therapies. Using untargeted liquid chromatography/mass spectrometry (LC/MS)-based metabolite profiling, we sought to characterize CSF for the discovery of *CLN2* disease-associated metabolic aberrations and potential biomarkers. Results uncovered 8 structurally-defined CSF metabolites that distinguish subjects with *CLN2* disease from control subjects and linearly correlate with *CLN2* disease severity - providing novel biomarkers with unprecedented potential for disease prediction, diagnosis, and therapeutic monitoring.

## Materials and Methods

### Human cohorts

All procedures and experiments were performed in accordance with relevant guidelines and regulations. This study was conducted under a research protocol approved by the Weill Cornell Medicine Institutional Review Board (IRB) and reviewed and approved with informed consent by all parents or guardians of the *CLN2* disease subjects. CSF of the normal control group of healthy individuals was obtained commercially (Horizon Pharma, Dublin, and BioIVT, Westbury, NY). A pilot study cohort of human subjects (n = 21) was stratified into 2 groups, comprised of healthy controls (n = 10) and *CLN2* disease cases (n = 11). An independent validation cohort was composed of an additional 6 control and 11 *CLN2* disease cases. The demographics of the study subjects are summarized in Supplementary Table S1. For the initial study, the 21 CSF sample cohort derived from 9 males and 12 females. The independent validation cohort comprised 5 males and 12 females. For metabolomic analysis, all samples were initially blinded with regard to medical histories.

The clinical *CLN2* disease severity was assessed using the WCLS score^11^, as specified in Supplementary Table S2. Disease severity was additionally characterized by an independent magnetic resonance imaging disease severity score (MRIDSS)^13^. Cerebrospinal fluid (CSF) samples were obtained by lumbar puncture and analyzed by LC/MS using untargeted metabolite profiling to identify small molecule biomarkers (50–1000 Da) that may distinguish *CLN2* disease subjects from healthy subjects, and correlate with WCLS- and MRIDSS-quantified disease severity. In attempt to validate initially identified disease biomarkers, CSF samples were collected from an independent cohort of 17 subjects, categorized as either normal (n = 6) or *CLN2* disease (n = 11). Those samples and subjects were assessed for biomarkers and disease severity identical to that of the initial study cohort (Supplementary Table S2).

### Reagents

LC-MS grade acetonitrile (ACN), isopropanol (IPA) and methanol (MeOH) were purchased from Fisher Scientific. High purity deionized water (ddH_2_O) was filtered from Millipore (18 MΩ.cm at 25 °C). OmniTrace glacial acetic acid and ammonium hydroxide were obtained from EMD Chemicals. Glycero-3-phosphoinositol was purchased from Echelon Biosciences Inc., N-acetylaspartylglutamic acid, ammonium acetate and all other chemicals and standards were purchased from Sigma Aldrich in the best available grade.

### CSF metabolite extraction and metabolomics data acquisition

CSF metabolites were extracted by addition of 1 part CSF to 9 parts iced (vol:vol) 70% acetonitrile in 30% ddH_2_O, containing 0.2% ammonium hydroxide. After brief vortexing, samples were centrifuged for 20 min at 14,000 × g and 4 °C to pellet precipitated proteins. A 3 µl aliquot of the resulting extract was subjected to untargeted metabolite profiling using LC/MS in both positive- and negative-ion monitoring mode. Randomization of the analyzed sample sequence was used to limit potential sample-order bias. To eliminate potential bias that may arise from day-to-day and batch-to-batch variation in extraction, chromatography and MS detection, a selection of CSF samples that were analyzed in the initial test set were newly reextracted (from frozen aliquots) and incorporated into the sample worklist for validation set analysis. Those duplicated samples were used for normalization and integration of both datasets.

### LC/MS metabolomics platform for untargeted metabolite profiling

An Agilent Model 1200 liquid chromatography system coupled to an Agilent 6230 time-of-flight (TOF) mass analyzer was used to analyze CSF extracts by LC/MS, as described previously^18,19^. Aqueous normal phase (ANP) gradient chromatography was performed using a diamond Hydride column (4 µm, 100 Å, 150 mm × 2.1 mm ID, Microsolv) for optimal separation of hydrophilic species. The mobile phases consisted of: (A) 50% isopropanol, containing 0.025% acetic acid, and (B) 90% acetonitrile with 5 mM ammonium acetate. To attenuate interference by contaminating metal ions with chromatographic separation and electrospray ionization, EDTA was added to the mobile phase at a final concentration of 6 µM. The following gradient was applied: 0–1.0 min, 99% B; 1.0–15.0 min, to 20% B; 15.0 to 29.0, 0% B; 29.1 to 37 min, 99% B. The sample injection volume was 3 µL, flow rate was 400 µL/min, and column temperature was 25 °C. Metabolites were quantified using an Agilent 6230 TOF MS with a dual electrospray ionization source, operating in positive- and negative-ionization modes, with a gas temperature of 300 °C, gas flow of 12 L/min, nebulizer pressure of 35 psi, capillary voltage of 3500 V, fragmentor voltage of 140 V and continuous reference mass correction at *m/z* 121.0509 and 922.0890 in positive-ion, and *m/z* 119.0363 and 966.0007 in negative-ion modes. Metabolite features were assigned from the raw data using MassHunter Profinder 8.0 (Agilent), considering an in-house accurate mass and retention time database derived from pure chemical standards^20–22^.

### Statistical methods

The MassProfiler Professional 13.0 software package (Agilent Technologies) was used to identify significant between-group differences for *CLN2* disease vs. control cases. Error bars represent standard deviation of the mean (SD). Welch’s t-tests, assuming unequal variances (p < 0.05), were used for statistical testing of between-group comparisons. Graphical analysis of results and Welch’s t-tests were performed using GraphPad Prism^19,21–24^. *CLN2* disease classification was performed using the proprietary open-source software Metaboanalyst 4.0 ^25^.

### Identification of differentially expressed metabolites

To assign structural identities to differentially expressed metabolites (p < 0.05) in *CLN2* disease subjects vs. healthy control individuals (Welch’s t-test, unpaired, unequal variance), LC/MS data were searched against an in-house Personal Compound Database and Library (PCDL) comprised of 806 accurate mass metabolite standards with defined formulae and chromatographic retention times. Differentially-expressed molecular features that were not found in our in-house database were initially compared against the *METLIN* Database (Agilent Technologies) and the Human Metabolome Database (HMDB; http://www.hmdb.ca). Potential structural matches were verified by comparison with pure metabolite reference standards that were analyzed on the same LC/MS platform to define chromatographic retention time and tandem mass spectrometry (MS/MS) fragmentation spectra at multiple collision energies (10, 20 and 40 eV).

## Results

### Untargeted profiling of CSF from subjects with *CLN2* disease

**Figure 1:**
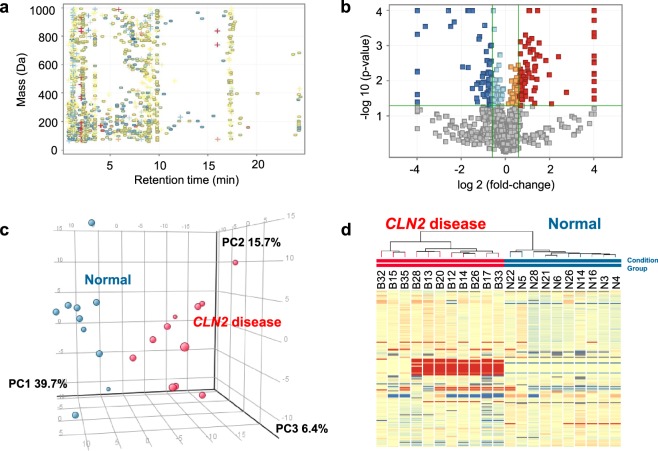
Untargeted metabolite profiling of CSF from *CLN2* disease subjects and normal controls. (**a**) Plot of retention time (min) versus monoisotopic mass. 1433 features that were found with >60% frequency in CSF samples in at least one group. The color coding represents the log (fold-change) compared to the group of healthy individuals (Normal) versus *CLN2* disease subjects (log FC −5 blue, 0 yellow,+5 red), shape coding represents the direction of regulation (+up, − down) in relation to the normal control group. (**b**) Volcano Plot depicting statistical analysis for 1433 observed entities, using an unpaired t-test unpaired (assuming unequal variance, Welch’s test), with p-value cut-off 0.05 and no correction for multiple hypothesis testing (blue/light blue, down-regulation in the *CLN2* disease vs Normal; red/orange, up-regulation in *CLN2* disease vs Normal; green lines (horizontal: p-value cutoff 0.05; vertical: Fold-change cutoff 1.5). Metabolite features that meet the criteria p < 0.05 and FC > 1.5 appear in red or FC < (−1.5) appear in blue. (**c**) Three-dimensional principle component analysis (PCA) plot of 257 significant differentially-expressed metabolite features, comparing the *CLN2* disease group vs. normal controls. PC1 accounted for 39.7% of the variation between the two groups and PC2 for 15.7%. (**d**) Unsupervised hierarchical cluster analysis (HCA), displaying the expression pattern of 158 significantly differentiated features (p < 0.05, FC > 1.5). Each column represents one biological sample and each horizontal line represents one metabolite feature sorted by retention time, from short (top) to long (bottom).

LC/MS metabolite profiling was performed as previously described utilizing aqueous normal phase chromatographic separation, coupled to time-of-flight MS^18,23,26^. Using this approach, the relative abundance of 794 positive ions and 581 negative ions in the range of 50–1,000 Da was quantified in ≥60% of samples from at least one group. Positive and negative ion features were combined and duplicate features were removed. The resulting 1,346 aligned molecular features were quantified in all CSF samples (Fig. 1a). In addition to the untargeted profiling approach, targeted metabolite profiling was performed by applying an in-house metabolite database of 806 endogenous metabolites. Using this approach, structural identities for 75 positive ions and 101 negative ions were detected that were initially not detected by the untargeted metabolite feature finding algorithm. Of these known metabolites, 36 were detected in both positive and negative ion mode. After removal of duplicated metabolites, a total of 140 defined metabolites resulted from the targeted metabolite profiling. Figure 1a summarizes the total of 1,433 metabolite features (combined from targeted and untargeted metabolite profiling resulting in 1,486 metabolite features; 53 duplicates form both untargeted and targeted profiling were removed) that underwent differential analysis and fold-change testing and after application of a frequency filter requiring that a metabolite feature is present in at least 60% of the samples of one sample group. A Welch’s t-test uncovered 257 significant differentially-expressed metabolite features, comparing *CLN2* vs. control groups (*p* < 0.05, Fig. 1b). Among these differential features, 158 were ≥1.5-fold increased (Fig. 1b, depicted in red) or ≥1.5-fold decreased (Fig. 1b, depicted in blue), compared to the control group. A principal component (PC) analysis was performed on 257 differentially expressed metabolite features and the plot of the first three components is shown in Fig. 1c. PC1, a weighted linear combination of metabolite features that contribute most to the in-between group differences, contained 39.7% of the variance between groups, while PC2 accounted for variance = 15.7%, and PC3 accounted for variance = 7.0%. It shows that metabolite features that account for PC1 discriminate the *CLN2* disease from the control group. Unsupervised Hierarchical Clustering Analysis (HCA) of the 158 metabolites with ≥1.5-fold changes (FC) from control (up-or-down) was performed to visualize major group differences, revealing a clear pattern of within-group metabolite expression similarities and between-group differences (Fig. 1d). These 158 unknowns, each represented by a horizontal tic in the HCA, were sorted by retention time. The unknown metabolites that presented the highest fold changes between *CLN2* disease group and controls were considered for structural identification.

### *CLN2*-disease specific CSF metabolite changes

The rationale for this study was the hypothesis that mutations in the *CLN2* gene would be associated with a distinct CSF metabolome that could be used to develop a biomarker panel for assessing disease severity. Furthermore, identified metabolite changes could potentially inform on pathological mechanisms of disease initiation and progression. Accordingly, the 158 metabolite features that distinguish *CLN2* disease-subjects from control subjects (p < 0.05, FC > 1.5) were further inspected for their potential as disease biomarkers. Each metabolite was characterized by an exact mass and retention time, as well as an isotopic distribution pattern. Based on these parameters, multiple identities could be structurally assigned by comparison with our in-house personalized compound database and library of 806 biologically relevant small molecules defined by monoisotopic mass, isotope abundance and spacing and chromatographic retention time with the given chromatographic method. Each database match was confirmed by reanalysis of the raw data. Notably, pyruvate, isoleucine, homocitric acid and N-acetylneuraminic were found to be 1.6–2.3-fold decreased in the *CLN2* disease group vs. control (Supplementary Table S3).

Many additional metabolite features were initially not identified from our in-house database and subsequently subjected to further structure elucidation efforts. Based on monoisotopic mass accuracy, isotopic spacing and isotope distribution patterns, Agilent ID browser software was used to specify empirical formulae and unknown compounds were tentatively matched against KEGG, HMDB and METLIN databases, followed by MS/MS-based structure confirmation based on molecular fragmentation patterns.

### Structural identification by MS/MS fragmentation

The remaining potential *CLN2* disease biomarkers were further assessed for structure identity. To that end, a representative CSF sample from the *CLN2* disease group was chosen to perform MS/MS fragmentation on a quadrupole time-of-flight (QTOF) mass spectrometer.

Among the 158 differentially-abundant metabolites, 26 were increased by >16 fold in the *CLN2* group and detected in over 60% of the *CLN2* disease samples and undetected in the control samples. Initial MS/MS fragmentation suggested the drug Levetiracetam as the origin of 14 out of those 26 metabolites (Supplementary Fig. S1). Disclosure of the medical history of the *CLN2* subjects confirmed that Levetiracetam and also Clonazepam was administered to >60% of the *CLN2* disease individuals. All 26 features were assigned to both drugs (as ionic and isotopic species) and consequentially excluded from consecutive MS/MS fragmentation experiments seeking structure elucidation. Other drugs or drug metabolites revealed by the medical history were not expected to be included among differentially-abundant metabolites because a requirement for metabolite consideration was detection in >60% of subjects, and no additional medications satisfied this criterion. Nonetheless, those drugs were surveyed separately in this cohort and indeed erased by the 60%-frequency filter.

**Figure 2:**
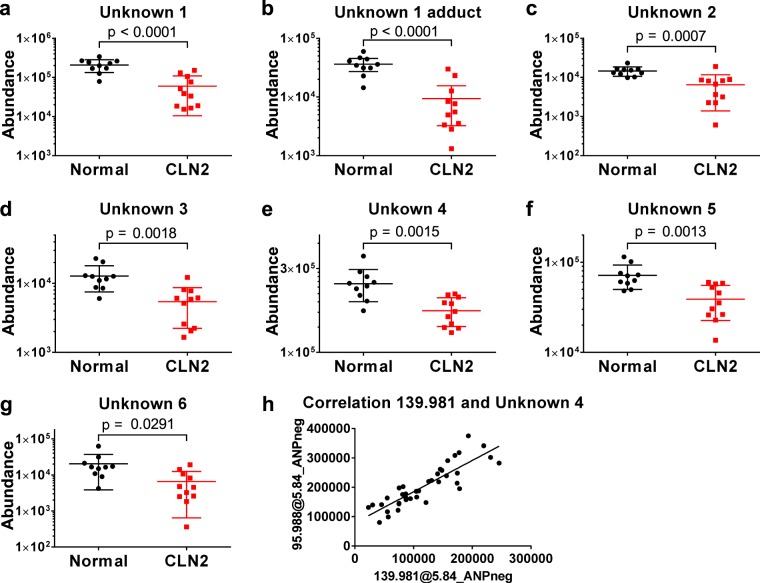
Unknown metabolites 1–6 differentiate *CLN2* disease subjects from normal controls. (**a**–**g**) MS signal abundances of unknown metabolites 1–6 are decreased in the *CLN2* disease group versus normal controls (p < 0.05, see Table 1 for details). Indicated signal abundances were quantified as areas under their cognate chromatographic peaks. h, Linear correlation of MS signal abundances of Unknown 4 with a co-eluting metabolite feature of 139.981 Da (Pearson correlation r > 0.85, p < 0.0001), indicating a relationship as a precursor and in-source fragment ion.

For further MS/MS-based structure identifications, several of the remaining 132 metabolites were evaluated with the criteria for consideration being FC > 2.0 and MS signal intensity >1,000 ion counts. Further, we prioritized the potential utility of yet-identified species based on their *CLN2* disease biomarker potential, based on their correlation with disease severity scores (MRIDSS and WCLS). Notably, the MRIDSS score developed by Dyke *et al*. ^13^ was previously shown to provide an objective measure for *CLN2* disease severity. Similar to WCLS^11^, the lower the MRIDSS the greater is the *CLN2* disease severity. Re-mining of the raw metabolomic data identified 29 metabolite features, corresponding to 6 unique unknown metabolites (represented as an array of ionic/isotopic species) that significantly correlate with the disease severity scores (p < 0.05, Pearson r > 0.5, Table 1). Notably, the abundance of each of these correlating metabolite features was significantly reduced in the *CLN2* disease cases, compared to controls (Fig. 2). A METLIN MS/MS database search suggested N-acetylaspartyl glutamate as the identity of Unknown 1, glycero-3-phosphoinositol as Unknown 2, methansulfonic acid as Unknown 4 whereas Unknowns 3, 5 and 6 initially remained unidentified.

**Table 1:** Linear correlation analysis of metabolite abundances in the test cohort with MRIDSS and WCLS values of each *CLN2* subject.

Compound Name	Detection mode	Mass (Da)	RT (min)	p (*CLN2* vs Control)	FC(*CLN2* vs Control)	Correlation Analysis				METLIN DB match
						MRIDSS		WCLS		
						Pearson r^2^	p-value	Pearson r^2^	p-value	
N-Acetyl-neuraminic Acid	ANPneg	309.104	6.34	9.5E-04	−2.21	0.2299	1.36-01	0.2838	9.2E-02	N-Acetyl-neuraminic Acid
N-Acetyl-neuraminic Acid	ANPpos	309.104	6.34	2.0E-03	−2.26	0.2007	1.7E-01	0.2684	1.0E-01	N-Acetyl-neuraminic Acid
Unknown 1	ANPneg	304.090	8.86	1.9E-04	−2.39	0.6039	4.9E-03	0.4587	2.2E-02	N-Acetylaspartyl-glutamic Acid
Unknown 1	ANPpos	304.090	8.86	1.8E-05	−4.47	0.5909	5.7E-02	0.3944	3.9E-02	N-Acetylaspartyl-glutamic Acid
Unknown 1 adduct	ANPneg	402.059	8.84	9.0E-05	−2.42	0.6104	4.5E-03	0.4871	1.7E-02	N/A
Unknown 1 fragment	ANPneg	286.084	8.84	4.3E-02	−14.5	0.5243	2.7E-02	0.4420	0.5E-02	N/A
Unknown 2	ANPneg	334.071	7.05	6.5E-02	−5.39	0.5256	1.8E-02	0.5664	1.2E-02	Glycero-3-phosphoinositol
Unknown 2	ANPpos	334.071	7.05	5.5E-02	−6.66	0.5988	8.6E-03	0.6750	3.6E-03	Glycero-3-phosphoinositol
Unknown 3	ANPneg	600.210	7.49	4.7E-05	−2.83	0.3771	4.4E-02	0.5024	1.5E-02	N/A
Unknown 4	ANPneg	95.988	5.84	1.0E-03	−1.38	0.3469	5.6E-02	0.3443	0.56E-02	Methane-sulfonic Acid
Unknown 5^a^	ANPneg	131.056	4.64	2.5E-03	−1.94	0.4235	3.0E-02	0.4012	3.6E-02	C_5_H_9_NO_3_
Unknown 6	ANPpos	733.544	7.69	1.2E-02	−1.45	0.3817	4.3E-02	0.4987	1.5E-02	N/A

^a^METLIN database search yielded a variety of possibilities for Unknown 5, e.g. aminolevulinic acid and cis/trans-4-hydroxyproline, which could be excluded by mismatching retention time to the in-house database entry. Formula matching of this metabolite is provided.

A Pearson correlation analysis was conducted with a two tailed p-value estimation.

### Validation of the *CLN2* metabolite signature

**Figure 3:**
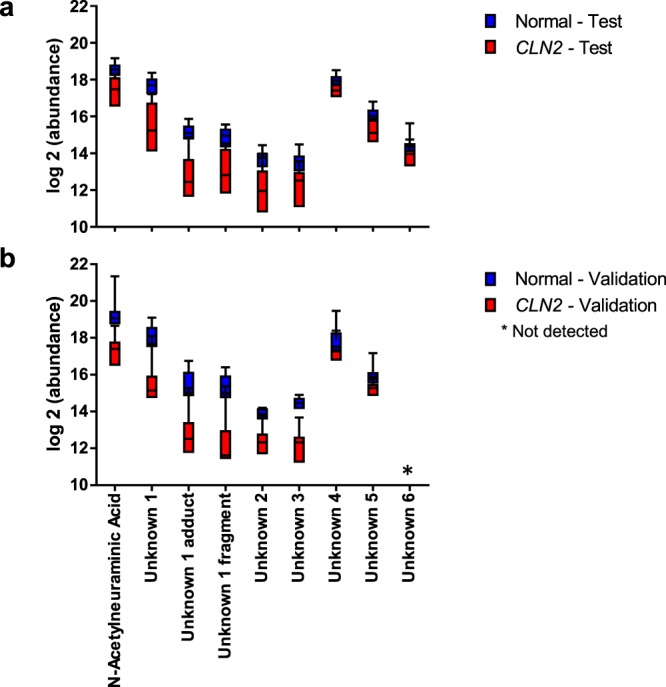
Validation of differential expression for selected metabolite features. (**a**) CSF metabolite profile of N-acetylneuraminic acid and Unknown 1–6 including Unknown 1 adduct and Unknown 1 fragment for *CLN2* and control individuals in ANPneg mode, except for unknown 6 which was only detected in the test cohort with positive ion monitoring (absence of this feature in the validation set (**b**)). (**b**) Abundance values of the metabolites of the validation set were normalized by the average fold change of B12, B13, B14, B15, B17, B20 and B26, as quantified on data acquisition days for both the initial case analysis and validation study cases.

An independent validation set analysis was performed in attempt to confirm the potential utility of identified *CLN2* disease metabolite biomarkers and disease signature. For this purpose CSF samples from an additional 10 *CLN2* disease subjects and 6 controls were extracted and profiled using the same LC-MS metabolomics platform as for the initial study groups. Since absolute MS signals can vary as a result of day-to-day and batch-to-batch variation, CSF aliquots of 7 *CLN2* disease cases from the initial sample group were concomitantly extracted and analyzed for data normalization. Notably, results of the validation study reconfirmed the initially identified *CLN2* disease metabolite biomarkers and signature (Fig. 3), including N-acetylneuraminic acid and Unknowns 1–5 that were found to correlate with the MRIDSS (Table 1). Unknown 6 was not detected in the validation cohort and thus excluded from further identification efforts (Fig. 3).

### Structural identification of Unknowns 1–5 by MS/MS fragmentation

MS/MS fragmentation experiments with pure reference standards for N-acetylaspartyl glutamate and glycerol-3-phosphoinositol, confirmed the identities of Unknown 1 and Unknown 2 (Supplementary Figs S2 and 3). MS/MS fragmentation of Unknown 3 resulted in a fragment that had the same *m/z* as N-acetylneuraminic acid, which initially was found to differentiate *CLN2* subjects from normal individuals (Supplementary Table S3). Unknown 3 could be attributed to N-acetylneuraminic acid dimer and confirmed by an MS/MS spectral match to a reference standard (Supplementary Fig. S4). The identity of Unknown 4 was suggested to be methansulfonic acid. Nevertheless, this metabolite is not known to be a CSF constituent, suggesting that it may be derived as an in-source fragmentation product of a precursor metabolite. Thus, a correlation analysis with co-eluting metabolites, sharing chromatographic retention times, was performed which yielded a linear correlation (Pearson r > 0.85, p < 0.0001) of Unknown 4 with a metabolite feature of 139.981 Da (Fig. 2h). A METLIN MS/MS database search resulted in the assignment of sulfoacetic acid as the parent metabolite for Unknown 4. Notably, in the original MRIDSS correlation analysis, sulfoacetic acid didn’t meet the p-value cutoff for significance of < 0.05 with an actual p-value of 0.0503. Unknown 5 was identified as N-acetylalanine by chromatographic retention time and MS/MS fragmentation match with a pure reference standard (Supplementary Fig. S5).

### Acetylated amino acids correlate with disease severity

Most of the above mentioned metabolite biomarkers (i.e., species with abundances that correlate with *CLN2* disease severity) carry an acetyl function (with the notable exception of glycero-3-phosphoinositol). We thus asked the question, if other acetylated metabolites can be found to correlate with disease severity. Targeted data remining of the acquired data for yet unidentified acetylated amino acids revealed levels of N-acetylserine and N-acetylthreonine to also be significantly attenuated in the *CLN2* disease group, versus control (Supplementary Fig. S6, further confirmed in the validation sample set). The identity of N-acetylserine was confidently confirmed by both chromatographic retention time and MS/MS spectra of a representative case extract and a reference standard.

### A *CLN2* biomarker signature in CSF

**Figure 4:**
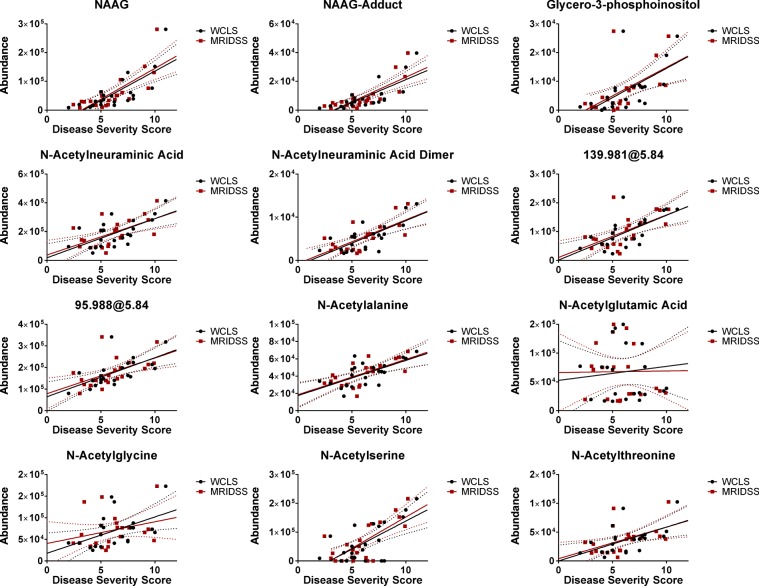
Identified *CLN2* disease-specific metabolite signatures linearly correlate with *CLN2* disease severity scores. Final Pearson correlation analysis and linear regression analysis of *CLN2* specific metabolite signatures and the magnetic resonance imaging disease severity score (MRIDSS) or the Weill Cornell LINCL score (WCLS) of all *CLN2* subjects (test and validation cohort). Relative metabolite abundances (ion counts, y-axis) are plotted against the disease severity scores (x-axis). Dotted lines represent the 95% confidence bands. Pearson r and p-values are summarized in Table 2.

Independent of the presented metabolomics study, all *CLN2* disease subjects underwent magnetic resonance imaging to develop a disease severity score based on MRI features (MRIDSS)^13^ – the lower the MRIDSS, the more severe is the disease. The same is true for the WCLS with a maximum score of 12 (Supplementary Table S2)^11^. Correlation analysis of combined CSF signatures, considering all *CLN2* disease subjects, was performed on the combined data of the initial test and validation cohorts. The results demonstrated the *CLN2* disease predictive efficacy in each of two independent data sets, acquired using an identical LC-MS platform. Notably, N-acetylneuraminic acid, N-acetylaspartylglutamic acid (and its adduct), glycero-3-phosphoinositol, N-actylneuraminic acid dimer, N-acetylalanine, sulfoacetic acid (characterized by its accurate mass 139.981 Da and its in-source fragment 95.988), N-acetylthreonine and N-acetylserine all significantly correlated with MRIDSS as well as with the WCLS scores. In contrast, N-acetylglycine and N-acetylglutamic acid did not exhibit a linear correlation with disease severity (Fig. 4, Table 2). Additionally, we performed a correlation analysis of signal abundances with the subjects’ gender and the subjects’ age. The Supplementary Fig. 7a shows the gender-specific signal abundances of the *CLN2* disease and the control group. There was no gender-specific significant difference within the group of the *CLN2* disease nor the controls subjects. As the MRIDSS and the WCLS linearly correlate with the age of the *CLN2* subjects (Supplementary Fig. 7b)^13^, a correlation of the defined CSF-biomarkers with age would not be surprising. In order to test if a correlation with the *CLN2* subjects’ age is specific to the condition, linear correlation analyses of both the control and the *CLN2* disease group were preformed (Supplementary Fig. 8, *CLN2* disease severity correlations in Table 2). Significant negative correlations of the *CLN2* disease severity biomarkers and age were obtained for N-acetylaspartylglutamic acid, glycerol-3-phosphoinositol, N-acetylneuraminic acid, N-acetylneuraminic acid dimer, N-acetylalanine, N-acetylserine and N-acetylthreonine. No such negative correlation was obtained for the group of the controls. Interestingly, for N-acetylneuraminic acid dimer and N-acetylglycine positive and thus opposite correlations with the age of the control subjects were obtained.

**Table 2:** Pearson correlation coefficients and p-values of *CLN2* signature metabolite abundances (normalized) and the MRIDSS, WCLS or age for all individuals (test and validation cohort).

Identification	Metabolite feature	WCLS		MRIDSS		Age (Months)	
		Pearson r	p-value	Pearson r	p-value	Pearson r	p-value
NAAG	Unknown 1	0.74	<0.0001	0.76	<0.0001	−0.7215	0.0002
NAAG-Adduct	Unknown 1 Adduct	0.75	<0.0001	0.78	<0.0001	0.1565	0.1976
Glycero-3-phosphoinositol	Unknown 2	0.6	0.003	0.57	0.0056	−0.5017	0.0174
NANA	N-Acetylneuraminic Acid	0.65	0.0009	0.59	0.0036	−0.3841	0.0776
NANA Dimer	Unknown 3	0.74	<0.0001	0.7	0.0003	−0.4997	0.0179
139.981	Unknown 4 parent	0.65	0.001	0.6	0.0029	−0.3848	0.077
95.988	Unknown 4	0.64	0.0015	0.57	0.006	−0.4181	0.0528
N-Acetylalanine	Unknown 5	0.66	0.0008	0.65	0.001	−0.5332	0.0106
N-Acetylglutamic Acid		0.11	0.6278	0.01	0.9585	−0.2377	0.2867
N-Acetylglycine		0.48	0.0224	0.28	0.2037	−0.37	0.0901
N-Acetylserine		0.67	0.0007	0.72	0.0001	−0.4748	0.0256
N-Acetylthreonine		0.55	0.0079	0.5	0.0175	−0.4243	0.0491

**Figure 5:**
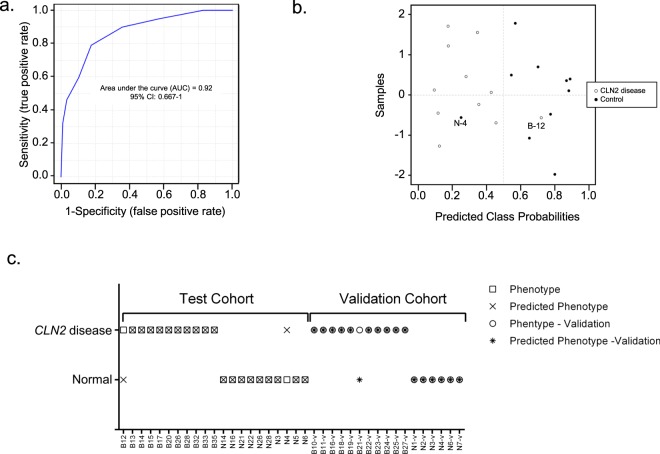
Characterization of a Supported-Vector-Machine (SVM) prediction model for the identification of *CLN2* based on 8 CSF-biomarkers. (a) Receiver-operating-characteristic (ROC)-analysis shows the sensitivity and specificity of the model with an area-under-the-curve (AUC) of 0.92 and (**a**) confidence interval (CI) of 0.667–1. (**b**) SVM-predicted class probabilities. A value of >0.5 leads to a *CLN2* disease classification, a value of <0.5 to a classification as a control. (**c**) Results of the predictive classification of the SVM-model for the test and validation cohort. B12 and N4 are misclassified in the test cohort, B21 is misclassified in the validation cohort.

Accordingly, the relative abundance of the afore-named metabolites offer telling biomarkers with the capability to shed light on *CLN2* disease severity and as a probe to monitor the efficacy of treatments. Additionally, recognition of these biomarkers could shed light on metabolic mechanisms that contribute to disease progression. In order to test the utility of all the presented 8 biomarkers, a supported-vector-machine (SVM)-prediction model based on the test cohort data was generated and applied to the validation data. The receiver-operation-characteristic (ROC) analysis resulted in an area under the curve (AUC) of 0.92 and a 95% confidence interval (CI) of 0.667–1. (Figure 5a,b) shows the predicted class probabilities for the test cohort and results in a mis-classification of B12 as control and N4 as *CLN2* disease case. Applying this SMV-prediction model, with a cutoff value of 0.5 to differentiate between *CLN2* disease and controls, to the validation cohort, results in only one misclassified *CLN2* disease case (Fig. 5c), B21-v. The cases B12 and B21-v were the subjects with the highest disease severity scores (B12: MRIDSS 9.06, WCLS 10; B21-v: MRIDSS 10.1, WCLS 11, Supplementary Table 2) and thus almost shows no symptoms.

## Discussion

The metabolic consequences of TPP1 deficiency/*CLN2* disease await comprehensive investigation. Indeed, despite identifying lipofuscin accumulation as a metabolic correlate of disease, neither the substrate of TPP1, nor why its deficiency causes neuronal cell death are known. By untargeted metabolite profiling, we identified highly-reproducible metabolic perturbations in the CSF of *CLN2* disease subjects that correlate strongly with disease severity and can therefore serve as a signature of disease status. Furthermore, these metabolic perturbations offer potential new insight into *CLN2* disease pathology and early neurodegeneration.

We performed LC/MS metabolite profiling of cerebrospinal fluid collected from a cohort of 22 *CLN2* disease and 16 control subjects. Correlation analysis with independently assessed disease severity parameters that resulted from clinical examination (WCLS) and magnetic resonance imaging (MRIDSS) revealed a panel of associated *CLN2* disease metabolite biomarkers. To our knowledge, this is the first comprehensive untargeted metabolite profiling study of CSF from children with *CLN2* disease. Importantly, a total of 8 metabolites were identified that each exhibit a linear correlation with both of the currently employed disease severity scores and thus are the first recognized CSF biomarkers for *CLN2* disease in children. *CLN2* subjects were treated with a personalized drug regimen that varied significantly from case to case in order to treat the conditions concomitant with the *CLN2* genetic defect according to their occurrence and severity. In this study, we chose healthy subjects as control group as control subjects without an underlying disease or condition that would not obfuscate the intended comparisons, but with matching drug regimens were impossible to recruit. To increase the confidence in the presented disease biomarker selection, we only considered CSF-metabolites that were reduced in concentration in the *CLN2* disease compared to the healthy control group and that thus positively correlated with current disease severity scores. As shown for Levetiracetam (Supplementary Fig. 1), a drug that was administered to 8 out of 11 *CLN2* disease cases in the test cohort, the untargeted algorithm detects drugs and their metabolites. One could argue that the presented CSF-biomarker changes may be caused by the medication the *CLN2* cases obtain rather than by the genetic *CLN2*-defect itself. To that end we performed a correlation analysis of the biomarkers with Levetiracetam and its co-dependent metabolite features for those *CLN2* cases that obtained this particular drug. A significant correlation which underlines that the drug concentration itself would directly be linked to the changes of the presented CSF-biomarkers was not obtained. This still leaves the question, if their changes are an adaptive response to the medication over time. In case of Levetiracetam, this possibility could be ruled out by the validation set, in which only one *CLN2* case received this drug. The other antiepileptic drugs of the *CLN2* disease cases, e.g. Valproic acid, Clonazepam and Clobazam, have different modes of action, which suggests that the changes in the CSF-metabolite profile would be different for each drug. Considering the variability that this introduces into the presented dataset, we argue that those drug-responsive changes are filtered out by the disease severity score correlation analysis.

Thus we suggest that the observed pattern of metabolite changes constitutes a fingerprint for *CLN2* disease and provide a new tool for effective monitoring of disease progression and response to therapies. Because *CLN2* disease subjects often receive afore mentioned anticonvulsant therapies that can markedly impact neurotransmitter levels in the CSF^27^, monitoring a single neurotransmitter or metabolite can be confounding. Here, we describe a pattern of metabolite changes that strongly correlate with the *CLN2* disease severity that is most likely disease-specific and unrelated to the medication received. These metabolites can all be detected and quantified using a single LC/MS metabolite profiling method and is expected to provide far superior knowledge of disease status than afforded by any single metabolite measurement.

The identities of *CLN2* disease-correlating metabolites may offer new information on disease pathology, as well as the metabolic consequences of TPP1 deficiency. Notably, all identified *CLN2* disease biomarkers in CSF, except glycerol-3-phosphoinositol, are acetylated species. The further the neurodegeneration progresses, the lower the CSF abundance of these metabolites. As acetate is the major cellular fuel, derived from both carbohydrate and lipid metabolism, the observed reduction of acetylated species in CSF raises the possibility of a disease-associated energetic deficit in neurons and/or glial cells. As diminished bioenergetics would predictably impair neuronal function and development, we suggest this defect to be a major driver of neurodegeneration. As a potential contributor to this bioenergetic defect, it is notable that TPP1 was shown to function as an exopeptidase in the sedolisin family, serving to cleave tripeptides from unsubstituted N-termini of specific neuropeptides^28^, including subunit C of mitochondrial ATP synthase^7^.

Tian *et al*.^29^ reported that TPP1 cleaves tripeptides from the unprotected N-terminus of polypeptides, generating new N-termini that can be available for potential acetylation. As those N-termini are either not present, or at low levels with TPP1 deficiency in *CLN2* disease, there is reduced capacity to generate additional acetylated peptide products which in consequence could diminish the levels of N-acetylated amino acids as proteolytic end-products. As the disease severity strongly correlates with the observed acetylated metabolites, it is conceivable that N-acetylaspartylglutamic acid, N-acetylalanine, N-acetylserine and N-acetylthreonine arise predominantly as downstream metabolites of the cleavage process initiated by TPP1. If so, proteolytic dysfunction in *CLN2* disease subjects may offer a basis for how these metabolites decrease over time, reflected in their CSF levels.

N-acetylaspartyglutamic acid (NAAG), the most abundant excitatory peptide neurotransmitter in the CNS, activates mGlu3 receptors and consequently inhibits the release of glutamate into the synaptic cleft^30^. The functional significance of its reduction in the CSF of *CLN2* disease subjects is yet unclear, nonetheless, this metabolite could serve as a readout for disease severity and progression. NAAG is hydrolyzed by glutamate carboxypeptidase II into N-acetylaspartate and N-acetylglutamate^31^ In contrast to a reduction in NAAG, the concentration of its downstream metabolite N-acetylaspartic acid (NAA) was not significantly changed in the CSF, whereas Worgall *et al*.^11^ and Dyke *et al*.^11,13^ describe reductions of this compound via whole brain MRI imaging. Interestingly, glutamic acid was observed to be slightly, but significantly (p < 0.05), reduced in the *CLN2* disease subjects’ CSF in both the test and validation cohort cohorts.

To our knowledge, the current literature only describes elevated levels of NAAG in Canavan disease, arising from a deficiency in aminoacylase 2, the enzyme principally responsible for the breakdown of N-acetylaspartate. Patients with free sialic acid storage disease (Salla disease), another recognized lysosomal storage defect, also exhibit increased CSF levels of NAAG and N-acetylneuraminic acid^32^. Additionally, low levels of NAAG in brain tissue and increased levels in CSF are associated with human amyotrophic lateral sclerosis (ALS)^33^. Interestingly, we find this metabolite to be significantly attenuated in *CLN2* CSF, and linearly correlating with disease severity. The cause of reduced NAAG levels await specification. However, since a combined reduction in levels of NAAG, N-acetylalanine, N-acetylthreonine and N-acetylserine hasn’t been previously recognized as associated with any inborn error of metabolism, we speculate that this pattern of change can serve as a unique identifier of *CLN2* disease.

Levels of N-acetylneuraminic acid (sialic acid) are known to be highest in the brain, associated with sialylated glycolipids (gangliosides). Polysialic acid was shown to be important in neuronal sprouting and plasticity^34^. Accordingly, low CSF levels of N-acetylneuraminic acid and its dimer in *CLN2* disease cases may contribute to neurodevelopmental delay and the neuroregression of afflicted patients.

In addition to acetylated species, sulfoacetic acid was found to be significantly diminished in CSF from *CLN2* disease cases, with levels that correlate inversely with disease severity. A slight reduction in taurine level is concomitant, but not correlating with the disease severity, suggesting that the taurine pathway in neuronal cells of *CLN2* disease cases may be perturbed. For drug-naïve patients with schizophrenic disorders, a reduction of CSF taurine has previously been described^35^.

Even though the precise functional roles of the downregulated biomarkers in *CLN2* disease subjects’ CSF is yet unclear, their identities have previously been associated with neurological disorders and thus may offer insight into TPP1 deficiency as a metabolic disease.

In summary, a NAAG reduction in CSF combined with lower levels of N-acetylneuraminic acid, N-acetylneuraminic acid dimer, N-acetylalanine, N-acetylserine and N-acetylthreonine as well as glycero-3-phosphoinositol and sulfoacetic acid, have not been detected in any other disease and thus offer a unique biomarker signature for *CLN2* disease diagnosis and prognosis. This pattern of change can be regarded as a metabolic fingerprint for *CLN2* disease with a molecular basis that demands elucidation.

## Electronic supplementary material

Supplementary Information

## Data Availability

The authors declare that all materials, data and associated protocols used in the present study will be available to readers without undue qualifications in material transfer agreements.
