# Supplementary material for: Untargeted Metabolite Profiling of Cerebrospinal Fluid Uncovers Biomarkers for Severity of Late Infantile Neuronal Ceroid Lipofuscinosis (CLN2, Batten Disease)
Source: Sci Rep. 2018 Oct 15;8:15229. doi: 10.1038/s41598-018-33449-0 (PMC6189193; doi:10.1038/s41598-018-33449-0)
Supplement: Supplementary file 1 — Supplementary Information [file 41598_2018_33449_MOESM1_ESM.pdf]

Untargeted Metabolite Profiling of Cerebrospinal Fluid Uncovers Biomarkers for Severity of Late Infantile Neuronal Ceroid Lipofuscinosis (*CLN2*, Batten Disease)

Miriam Sindelar[1], Jonathan P. Dyke[2], Ruba S. Deeb[3], Dolan Sondhi[3], Stephen Kaminsky[3], Barry E. Kosofsky[4], Douglas Ballon[2,3], Ronald G. Crystal[3] and Steven S. Gross[1]

[1]Department of Pharmacology, Weill Cornell Medicine, New York, New York
[2]Department of Radiology, Weill Cornell Medicine, New York, New York
[3]Department of Genetic Medicine, Weill Cornell Medicine, New York, New York
[4] Department of Pediatrics, Weill Cornell Medicine, New York, New York

Supplementary Information

Structural identification of administered drugs by MS/MS fragmentation. Initial selection criteria for MS/MS fragmentation studies included an apparent FC >2 (increase or decrease), chromatographic retention time (RT) > 2 min (to avoid interfering co-eluting metabolites) and an MS signal intensity >1000 counts. Importantly, the larger the between-group fold-change, the greater the potential to serve as a reliable biomarker. Thus, in a first series of MS/MS fragmentation experiments, we sought to identify metabolite features that were abundantly detected in only one group. Several metabolite features fulfilling this criterion showed large signal intensities and a log FC >16 in *CLN2* vs. the control group (i.e., essentially undetected in controls) with chromatographic RT of 2.0 – 2 .2 min. Because these features were essentially undetectable in the controls (except for feature 854.5403@2.01, with a clearly detected signal in only one of the control cases) they offer in theory ideal biomarker qualities (Supplementary Fig. S1a). The abundance relationship of the co-eluting metabolite features in the 2.00-2.02 min range with ANP chromatography (positive-ion monitoring MS) suggested co-dependency of some species as in-source fragments and/or adducts. Notably, none of the control samples contained any of these metabolites, despite observation in 8 out of 11 *CLN2* disease samples.

One metabolite from this group with an assigned singly-protonated mass of 170.1049 was chosen for MS/MS fragmentation, concurrently represented as mass 192.0871, presumably a sodium adduct of the same molecular species. Surprisingly, three *CLN2* disease CSF samples did not possess these features. In consequence, we considered the possibility that these molecular species arise from some medication that most, but not all, *CLN2* disease cases were receiving. As noted in the *Methods* section, the study was initially blinded for medication history of the cases. A potential ”hit” in the METLIN database for the monoisotopic mass of 170.1049 was the antiepileptic drug, Levetiracetam. Disclosure of the medical history of the *CLN2* subjects confirmed that Levetiracetam was a common medication received by all *CLN2* disease individuals, except for cases B15, B32 and B35 in which this species was undetected. Several detected lower molecular mass features could be attributed to in-source fragments of this species, in accord with the chemical fragmentation pattern depicted in Supplementary Fig. S1b. To further confirm that coeluting metabolite features (Supplementary Fig. S1a) with assigned lower molecular masses arose as in-source fragments of Levetiracetam, a representative CSF sample was analyzed with a QTof to assess positive- and negative-ion mode MS/MS fragmentation at 10, 20 and 40 eV collision energy with a parent *m/z* 171.1128 (Supplementary Fig. S1c). Results confirmed that several co-eluting higher molecular mass features (adducts, and multiply charged metabolite features) were indeed attributable to administered Levetiracetam in drug-treated *CLN2* disease cases. Uncovering Levetiracetam as a differentially expressed small molecule in *CLN2* disease versus control CSF samples provided confidence in our use of untargeted metabolite profiling platform for detection and quantification of significant CSF metabolite changes with potential to confidently report on *CLN2* disease.

Confirmation of potential *CLN2* disease biomarker structures by MS/MS fragmentation analysis. MS/MS fragmentation experiments were performed with a QTof for unknowns specified in Table 2. As Unknown 6 was not observed in the validation set, it was excluded from identification. Unknown 1-3 and 5 were structurally characterized by MS/MS fragmentation experiments in positive and negative ion monitoring. (Supplementary Fig. S2-5)

Unknown 1 – N-Acetylaspartylglutamic Acid. Unknown 1 shared the retention time with a metabolite feature characterized by an *m/z* of 401.0495 in negative ion monitoring (Supplementary Fig. S2a). Linear regression analysis of both signal abundances confirmed their close relationship with an r^2 of 0.97, indicating their signals originate from the same parent metabolite. (Supplementary Fig. S2b) The feature of *m/z* 401.0495, which we infer to be a phosphate adduct of Unknown 1, was absent in positive ion monitoring. For Unknown 1, the METLIN database matched N-acetylaspartyl glutamic acid (NAAG), the most abundant peptide neurotransmitter in the mammalian brain[26]. Comparison of the MS/MS-fragmentation of a representative CSF extract (B12) with a pure NAAG standard solution confirmed the identity of Unknown 1 by matching both retention times and MS/MS spectra at 10 and 20 eV collision energy (Supplementary Fig. S2c). Figure 4, Panel D shows a fragmentation pattern of NAAG assigning chemical formula to the major fragments for the observed MS/MS spectra.

Unknown 2 – Glycero-3-phosphoinositol. Unknown 2 was identified by MS/MS fragmentation experiments as glycero-3-phosphoinositol, by comparison with a pure reference standard. Notably, both chromatographic retention time and the MS/MS spectra at 10 and 20 eV in negative ion monitoring matched (Supplementary Fig. S3a), confirming the suggested METLIN

database ID. Supplementary Figure S3b depicts a fragmentation pattern that reconciles with the experimentally observed fragment ions.

Unknown 3 –N-Acetylneuraminic acid dimer (disialic acid). MS/MS fragmentation of Unknown 3 at 7.49 min in positive ion detection mode yielded an *m/z* of 310.11 as a major fragment at 0 and 10 eV, that disappeared at higher collision energies of 20 and 40 eV. MS/MS experiments of an N-acetylneuraminic acid standard (*m/z of* 310.1133, RT 6.34 min) confirmed this behavior for N-acetylneuraminic acid in accord with this species as a functional group in Unknown 3. As both metabolite features significantly differ in their chromatographic retention times, a relationship as in-source fragments or adducts with mobile phase additives could be excluded. Notably, the assigned molecular mass of Unknown 3 matched the O-glycosylated dimer of N-acetylneuraminic acid, which was commercially available. MS/MS fragmentation of a standard solution at 10 and 20 eV collision energy affirmed that Unknown 3 was N-acetylneuraminic acid dimer (disialic acid, Supplementary Fig. S4a) with major fragmentation products confidently assigned (Supplementary Fig. S4b).

Unknown 4 – Sulfoacetic Acid. MS/MS fragmentation experiments in negative mode of the potential precursor of Unknown 4 (139.981 Da) revealed both Unknown 4 and *m/z* 79.9574 (ascribed to SO_3^-) as major fragments of this metabolite and the identity of this species origin as sulfoacetic acid was highly suspect. This metabolite is a downstream product of taurine and hypotaurine metabolism. Support for assignment as sulfoacetic acid was provided by a targeted remining of the data for metabolites in the taurine biosynthetic pathway, revealing taurine to be significantly diminished in CSF from *CLN2* cases. Notwithstanding, it was found to be modestly reduced and failed to meet the FC threshold of > 1.5 for consideration as a *CLN2* biomarker.

Unknown 6 – N-Acetylalalanine. As stated earlier, a METLIN database search scored N-acetylalanine as a likely identity for Unknown 6, eliminating alternative isobaric identities such as 5-aminolevulinic acid and hydroxyl-proline based on observed differences in chromatographic retention times. A pure N-acetylalanine standard solution confirmed a retention time match with Unknown 6. An MS/MS spectrum of a representative control sample (N-4) was compared to the MS/MS spectrum of the N-acetylalanine standard revealed *m/z* 88.0404 in negative ion monitoring as a major common fragment (Supplementary Fig. S5a). This fragment is attributed to collision-induced loss of an acetyl functionality (Supplementary Fig. S5b).

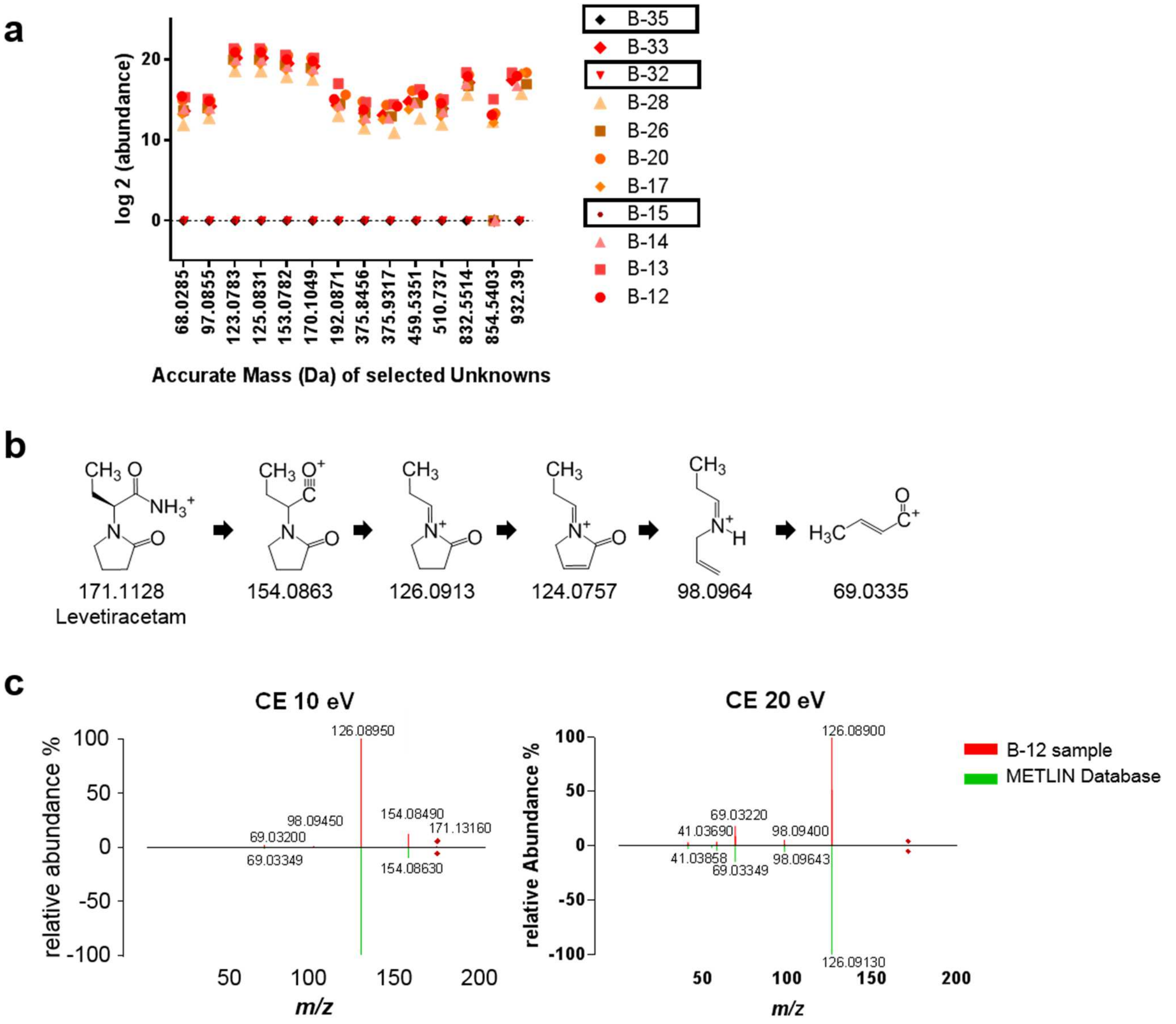

Supplementary Figure S1: Levetiracetam gives rise to a group of Unknowns that differentiates the *CLN2* disease group from normal controls.

a, Log 2 normalized metabolite abundances in *CLN2* CSF extracts with a shared chromatographic retention time of 2.01 min recorded in positive ion monitoring (ANPpos)X-axis: monoisotopic mass (Da) calculated from detected *m/z*. The presented metabolite features were not detected in the controls (except for feature 854.5403@2.01, with a clearly detected signal in only one control cases) and B15, B33 and B35.

b, Proposed in-source fragmentation pattern of levetiracetam (170.10553 Da).

c, MS/MS fragmentation experiments of m/z 171.1118 as precursor ion and 10, 20 or 40 eV collision energy. X-axis: *m/z* of proposed in source fragments (structures below). The signals are absent in all the normal controls (N4 to N28) and in the *CLN2* samples of the patients B15, B32 and B35. Only the feature 854.5403@2.01 is observed in the B32 and missing in B28 sample.

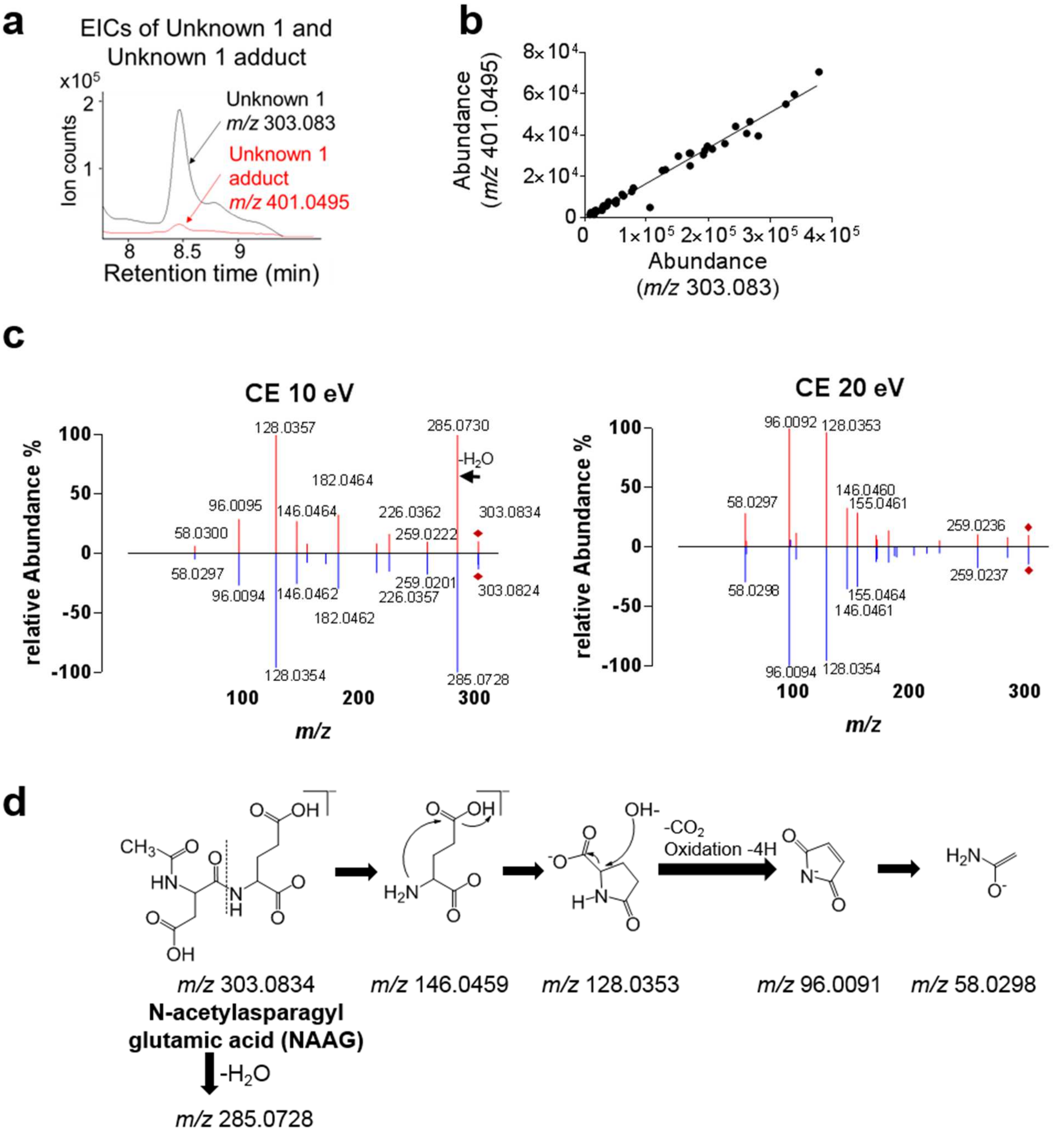

Supplementary Figure S2: MS/MS fragmentation experiments confirm N-Acetylaspartyl glutamic acid as the identity of Unknown 1 and a related adduct.

a, Confirmation of adduct formation in a standard solution of NAAG. Similar retention times for m/z 303.0834 (NAAG) and its proposed adduct (M 402.059 Da).

b, Linear regression analysis of the abundances of Unknown 1 and Unknown 1 adduct (r^2 = 0.97).

c, MS/MS spectra at 10 eV collision energy of Unknown 1 in a B12 extract in comparison with a 100 nM NAAG standard solution.

d, Possible fragmentation pattern of NAAG, structures assigned to the major fragments at 10 eV collision energy.

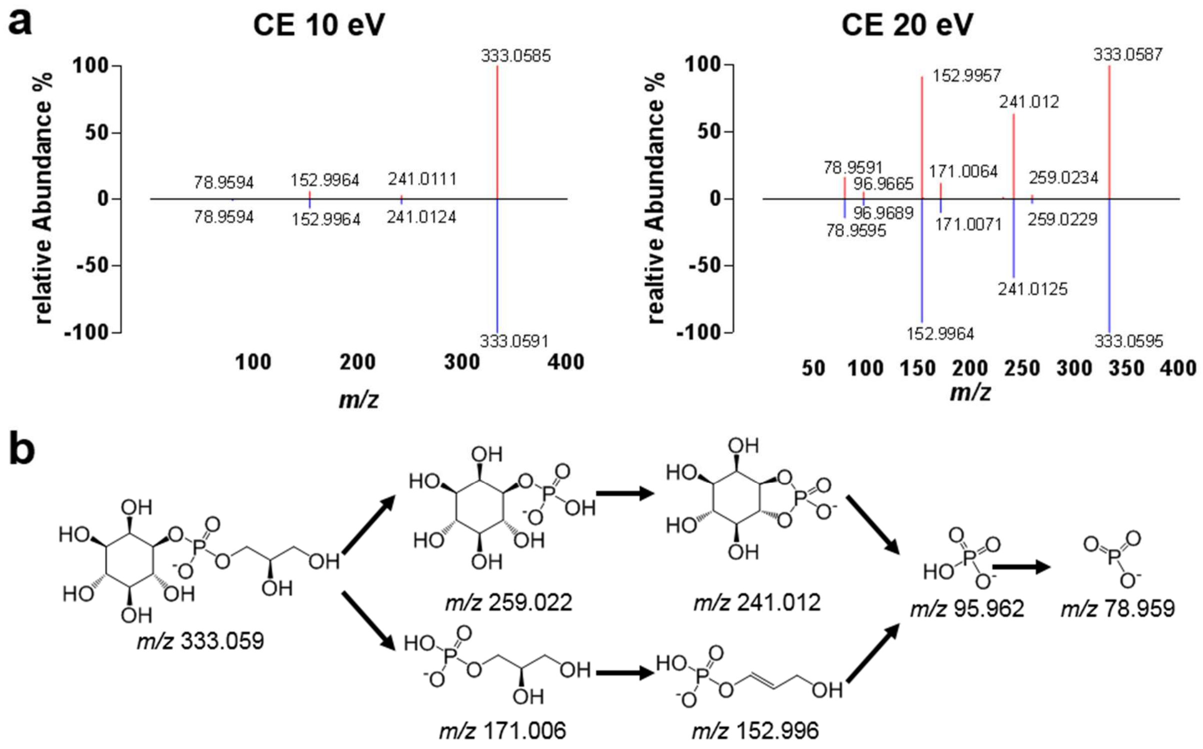

Supplementary Figure S3: MS/MS fragmentation experiments identify Unknown 2 as glycero-3-phosphoinsolitol.

a, Comparison of an extracted MS/MS spectrum of Unknown 2 in a representative B12 extract (red, upper panel) and a pure 100 nM glycero-3-phosphoinositol standard (blue, lower panel) at 10 eV and 20 eV collision energies, in negative ion monitoring. Shown are the relative abundances, scaled to the largest peak in the extracted MS/MS spectrum.

b, Possible fragmentation pattern of Glycero-3-phosphoinositol in negative ion monitoring. Molecular formula are assigned to the major fragments at 10 eV and 20 eV collision energy.

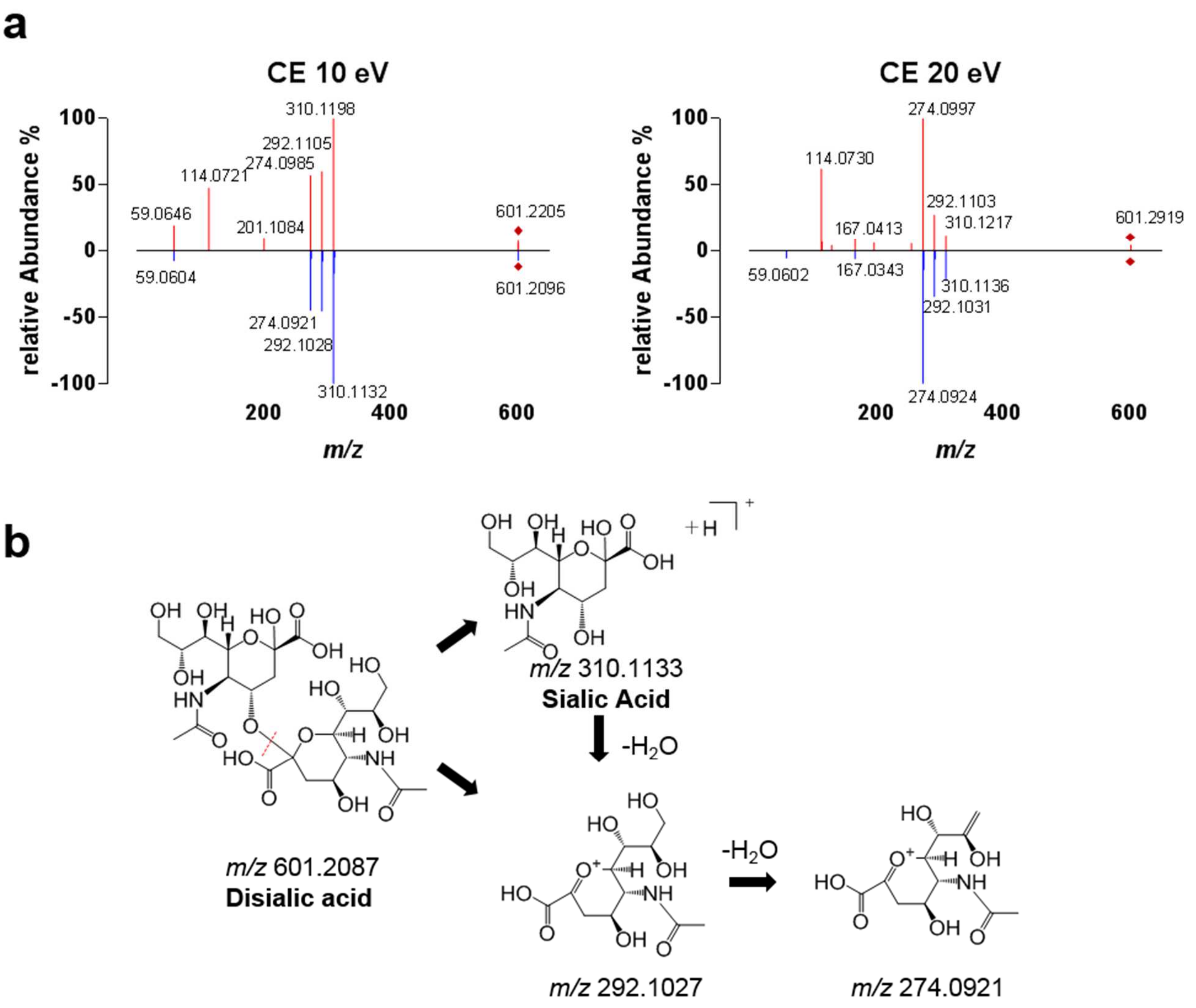

Supplementary Figure S4: MS/MS fragmentation experiments identify Unknown 3 as N-acetylneuraminic acid dimer (disialic acid).

a, Comparison of an MS/MS spectrum of Unknown 3 in a representative B12 extract and a pure N-acetylneuraminic acid dimer standard at 10 and 20 eV collision energies (positive ion monitoring). B12 data has been assessed on a Qtof 6550, the N-acetylneuraminic acid dimer standard data on a Qtof 6538. *m/z* 114.0721 is suggested to be a fragment that only occurs in the Qtof 6550. Relative abundances scaled to the base peak of the extracted MS/MS spectrum are shown.

b, Possible fragmentation pattern of N-acetylneuraminic acid dimer in positive ion monitoring. Molecular formula are assigned to the major fragments at 10 eV collision energy, one of which is the $[M+H]^+$ signal of N-acetylneuraminic acid at *m/z* 310.1133.

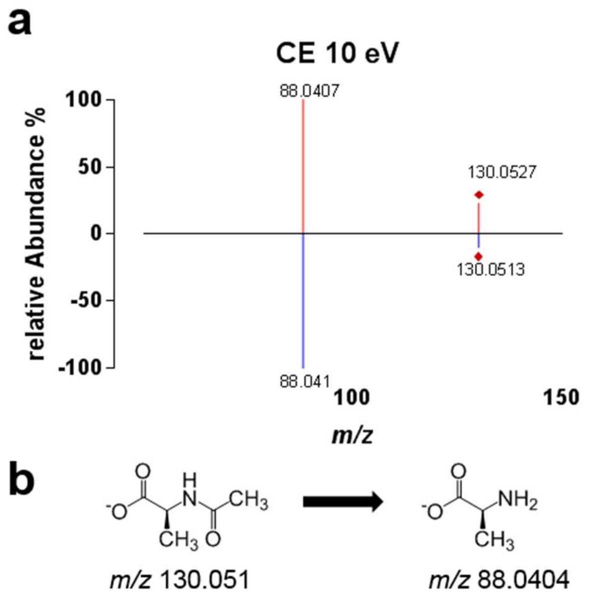

Supplementary Figure S5: MS/MS fragmentation experiments identify Unknown 6 as N-acetylalanine.
a, Comparison of an MS/MS spectrum of Unknown 6 in a representative N-4 extract and a pure n-acetylalanine standard.
b, Possible fragmentation pattern of N-acetylalanine in negative ion monitoring. Structural formula of the major fragment at 10 eV collision energy.

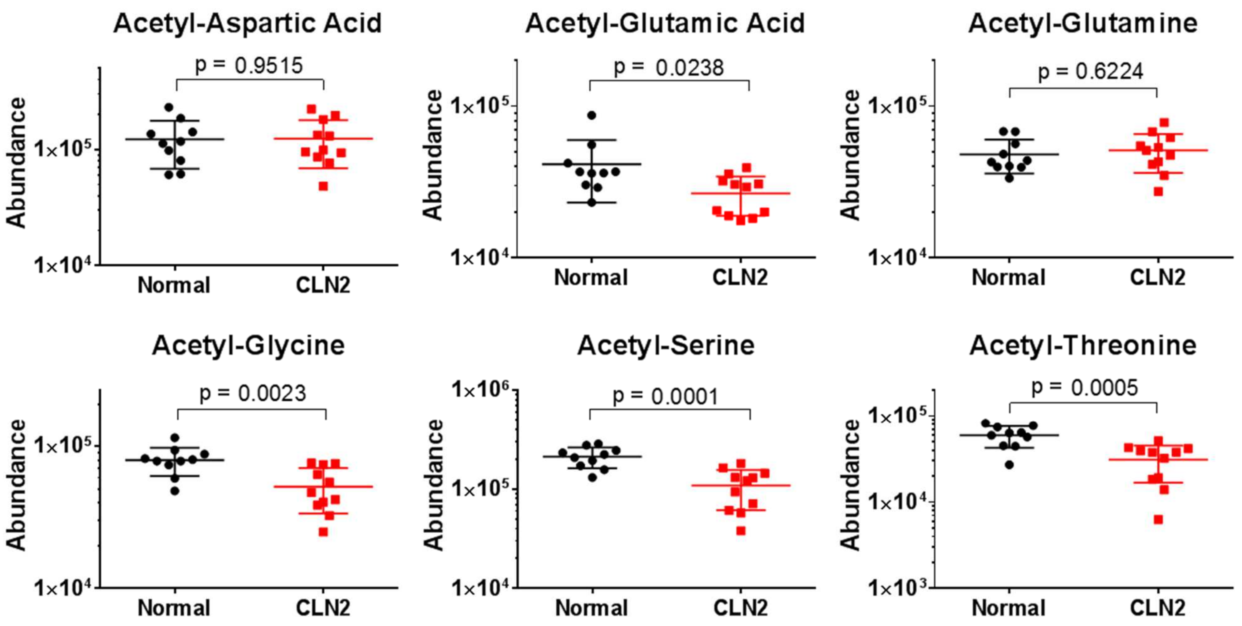

Supplementary Figure S6: N-acetylated amino acids distinguish *CLN2* from controls in the test data set. Shown are the relative abundances (ion counts) of the acetylated amino acids acetyl-aspartic acid, acetyl-glutamic acid, acetyl-glutamine, acetyl-glycine, acetyl-serine and acetyl-threonine recorded by LC-MS analysis in negative ion monitoring.

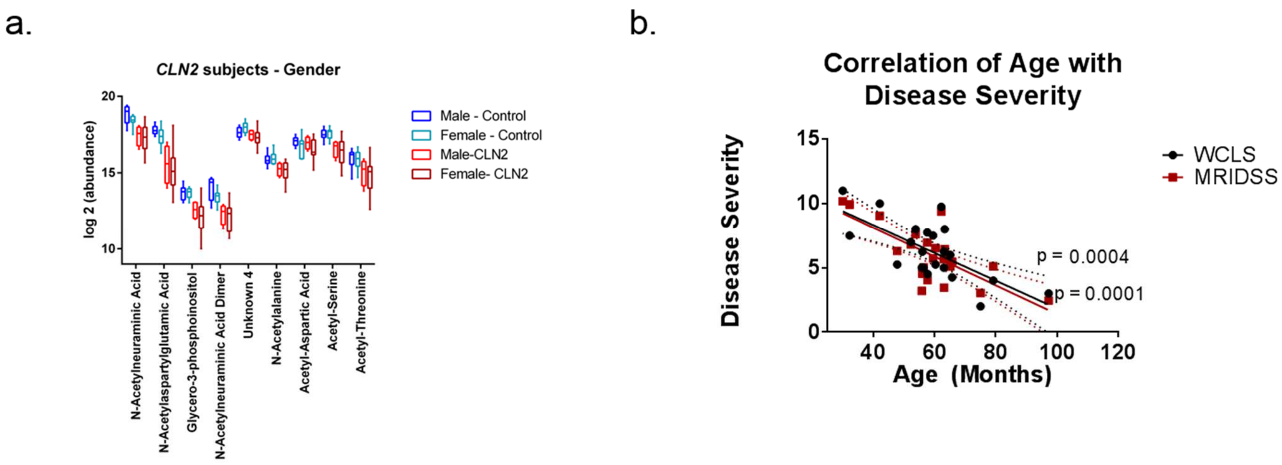

Supplementary Figure S7: CSF-biomarker Correlation Analysis with Gender. a. Signal abundances of CSF-disease severity biomarkers devided by gender. No significant difference was found according to a subject's gender group. b. A significant linear correlation of age and the MRIDSS and WCLS was found. The disease severity progresses with an individual's age.

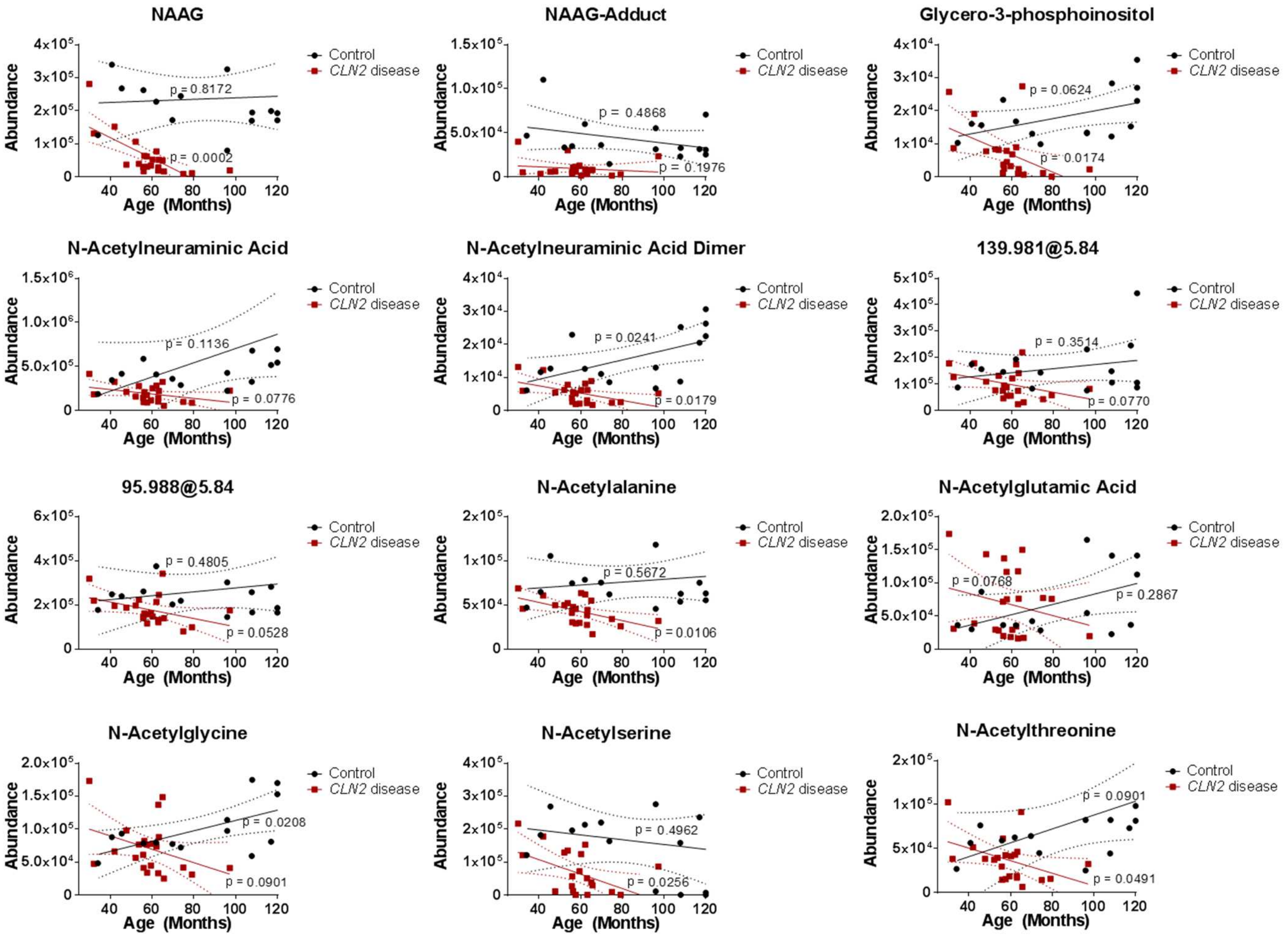

Supplementary Figure S8: Linear Correlation Analysis of the CSF-biomarker signal abundances and the age of normal control individuals and *CLN2* disease subjects.

Supplementary Table S1. Human Subject Demographics.

	**Normal**	**Batten/*CLN2***
**Test Cohort**		
Number of subjects	10	11
Gender	4M, 7F	5M, 5F
Age (months)	58.6 ± 15.5	70.3 ± 27.2
**Validation Cohort**		
Number of subjects	6	11
Gender	4M, 2F	1M, 10F
Age (months)	106 ± 17.5	59.2 ± 12.5

M = male, F = female

Supplementary Table S2. Individual subjects' age (months), gender (M = male, F = female), Weill Cornell LINCL Scale and Magnetic Resonance Imaging Disease Severity Score (MRIDSS). N# define normal control subjects, B# define *CLN2* patients. v indicates the validation cohort.

Case Number	Age (months)	Gender	MRIDSS	Weill Cornell LINCL Scale
B12	42.1	M	9.06	10
B13	52.2	F	6.82	7
B14	53.8	M	7.62	8
B15	32.2	M	9.93	7.5
B17	65.7	F	5.47	4.25
B20	97.3	F	2.45	3
B26	60.3	F	6.53	5.25
B28	63.0	F	5.67	5
B32	62.2	F	9.40	9.75
B33	56.0	M	4.54	6.25
B35	59.5	F	5.73	7.5
N3	117	M	N/A[1]	N/A[2]
N5	96.0	M	N/A[1]	N/A[2]
N4	55.9	M	N/A[1]	N/A[2]
N6	73.8	M	N/A[1]	N/A[2]
N14	40.8	F	N/A[1]	N/A[2]
N16	45.5	M	N/A[1]	N/A[2]
N21	62.0	F	N/A[1]	N/A[2]
N22	34.1	F	N/A[1]	N/A[2]
N26	69.8	F	N/A[1]	N/A[2]
N28	107.8	F	N/A[1]	N/A[2]
B10v	65.1	F	5.10	6
B11v	63.3	F	6.44	8
B16v	55.8	F	3.21	5
B18v	47.8	F	6.30	5.25
B19v	63.1	F	3.46	6.25
B21v	30.0	F	10.19	11
B22v	56.4	M	5.03	5
B23v	79.2	F	5.12	4
B24v	57.6	F	6.97	7.75
B25v	57.7	F	4.05	4.5
B27v	75.0	F	3.05	2
N1v	120	M	N/A[1]	N/A[2]
N2v	120	M	N/A[1]	N/A[2]
N3v	108	M	N/A[1]	N/A[2]
N4v	96	M	N/A[1]	N/A[2]
N6v	120	F	N/A[1]	N/A[2]
N7v	72	F	N/A[1]	N/A[2]

[1] Normal controls were not analyzed by MRI indicated by N/A. Maximum score of 12 indicates healthy status.
v indicates samples of the validation cohort
[2] The normal control CSF samples were purchased and individuals were not examined applying the Weill Cornell *LINCL* score. The maximum of 12 represents the healthy status with no cognitive or motoric impairments.
M = male, F = female

Supplementary Table S3: Differentially-expressed metabolites in *CLN2* disease vs. control subjects identified from in-house metabolite database search of the test cohort data.

Compound Name	Detection mode	Mass (Da)	Retention Time (min)	p-value (*CLN2* vs. Control)	Fold Change (*CLN2* vs. Control)	Regulation (*CLN2* vs Control)
Pyruvate	ANPneg	88.015	0.86	1.77E-02	-1.6	down
N-Acetyl-neuraminic acid	ANPneg	309.104	6.34	6.36E-04	-2.3	down
Isoleucine	ANPneg	131.090	7.20	3.35E-02	-1.7	down
Homocitric acid	ANPneg	206.042	8.57	4.66E-02	-1.7	down
